# Gastric Stump Cancer: More Than Just Another Proximal Gastric Cancer and Demanding a More Suitable TNM Staging System

**DOI:** 10.1155/2013/781896

**Published:** 2013-09-16

**Authors:** André Costa-Pinho, J. Pinto-de-Sousa, José Barbosa, J. Costa-Maia

**Affiliations:** ^1^Department of General Surgery, Centro Hospitalar S. João, Alameda Prof. Hernani Monteiro, 4200-319 Porto, Portugal; ^2^University of Porto Medical School, Alameda Prof. Hernani Monteiro, 4200-319 Porto, Portugal; ^3^Institute of Molecular Pathology and Immunology of the University of Porto (IPATIMUP), Rua Dr. Roberto Frias, 4200-465 Porto, Portugal

## Abstract

*Background*. Considerable controversy persists about the biological behavior of gastric stump cancer (GSC). The aim of this study is to clarify if this cancer is just another proximal gastric cancer or if it emerges as a distinctive clinicopathologic entity. *Methods*. This review of a prospectively collected gastric cancer database identified 73 patients with GSC in a single institution between January 1980 and June 2012 and compared them with 328 patients with proximal gastric cancer (PGC) and 291 patients with esophagogastric junction cancer (EGJC). *Results*. Patients with GSC were predominantly males. Eighty-three percent of GSC penetrated the subserosal or the serosal layers. The median number of lymph nodes retrieved in GSC patients was significantly lower than in PGC patients or in EGJC patients. Cumulative survival curves were not different between GSC, PGC, or EGJC patients. Unlike that observed in PGC and in EGJC, no significant differences in cumulative survival according to the TNM staging system were observed in GSC cases. *Conclusions*. The outcome of patients with GSC displayed significant differences when compared to those with other proximal gastric cancers concerning the lack of survival association with the TNM staging system. Therefore a more suitable staging system should be designed for these unique cancers.

## 1. Introduction

Gastric stump cancer (GSC), first described by Balfour in 1922 [1], is defined as a carcinoma occurring in the gastric remnant after partial gastric resection for peptic ulcer disease (PUD). Medical therapy has displaced partial gastrectomy in the treatment of PUD, but the incidence of GSC, reported to range from 1 to 8% [2, 3], has not declined linearly because of the long latency period required to carcinogenesis.

Gastric stump carcinogenesis has been tightly linked to environmental changes [4, 5] created by previous partial gastric resection that induces chronic damage to the remnant mucosa. These factors can hardly be associated with other types of proximally located gastric cancers.

Viste et al. [6] compared GSC patients with other gastric cancer patients and found differences in age, gender, stage distribution, resectability rates, and operative procedures; postoperative mortality and survival rates were similar. Many series from the literature either evaluated a scarce number of cases or included patients after partial gastric resection for cancer; therefore, available data reports highly inconstant results regarding pathology, treatment, and outcomes for these patients. Consequently, both biological behavior and optimal treatment are still controversial, and there is no clear consensus to consider GSC as just another proximal cancer or as a distinctive entity. 

This study is primarily aimed to assess if previous gastric resection for PUD influences the clinicopathologic features of GSC. To answer this question the present study has two specific objectives: (1) to define the clinicopathologic and prognostic factors of GSC and (2) to compare these factors with those observed in patients with proximal gastric cancer (PGC) and esophagogastric junction cancers (EGJC).

## 2. Material and Methods

GSC was defined as adenocarcinoma occurring in the gastric remnant at least 5 years after partial gastric resections for PUD. PGC was defined as adenocarcinoma originating in the proximal third of nonoperated stomach, and EGJC was defined as adenocarcinoma of the distal esophagus and proximal stomach, with the tumor center located within 5 cm above to 5 cm below the anatomic EGJ. Cases of metachronous adenocarcinomas and those of early recurrences of primary cancers were excluded from this study.

Data on patients admitted to our tertiary center with the diagnosis of upper gastrointestinal cancer, between January 1980 and June 2012, were recorded in an electronic prospective database that was queried for patients with GSC, PGC, and EGJC. 

Total gastrectomy extended to the distal esophagus, and splenectomy via left thoracophrenolaparotomy was the most frequently performed operation in EGJC patients. Total gastrectomy was performed in PGC patients. GSC patients were submitted to a completion gastrectomy either via an abdominal approach or through a left thoracophrenolaparotomy. D2 lymphadenectomy (removal of perigastric nodes and nodal tissue along the main branches of the celiac axis) plus omentectomy was the standard in these patients. En bloc splenectomy and/or extended resection of the jejunum were performed in selected patients with suspected nodes in the splenic hilum and/or in the mesojejunum, respectively. 

Several clinical and pathological parameters were evaluated. Macroscopic appearance was classified into fungating and ulcerofungating, ulcerated and ulceroinfiltrative, or infiltrative according to criteria previously described [7]. Hematoxylin-and-eosin-stained sections were used to categorize tumours according to Lauren [8] as intestinal, diffuse, or unclassified types. Venous invasion, evaluated in orcein-stained sections, was scored as absent or present. Lymphatic vessels invasion was also scored as absent or present.

Residual tumour status was defined using the standard R-classification: R0, no residual tumour; R1, microscopic residual tumour; R2, macroscopic residual tumour.

Carcinomas were staged according to the seventh edition of the American Joint Committee on Cancer TNM Staging Classification for Gastric Cancer [9].

The number of lymph nodes harvested, those positive for metastasis, and the ratio between the number of positive and retrieved lymph nodes (categorized into two groups considering the cut-off value of 20%) were recorded.

Only patients submitted to R0 resections were considered in the analysis of cumulative survival. Survival data was obtained from the Cancer Registry of our institution, with a followup of 99.2% of the patients.

All statistical analyses were performed using IBM SPSS Statistics, version 19.0 (Chicago, IL, USA, http://www.spss.com/). Distributions were compared by Chi-square test or by Fischer's exact test whenever appropriate. Continuous variables were compared by the Kruskal-Wallis nonparametric test. Cumulative survival curves were obtained by the Kaplan-Meier method [10] and compared using the log rank test. The Cox regression stepwise approach (proportional hazard model) was used to identify prognostic factors [11]. Significance was assumed for *P* values under 0.05.

## 3. Results

Between January 1980 and June 2012, 1497 patients were admitted to our tertiary surgical center with histologically diagnosed gastric cancer: 73 GSC patients, 328 PGC patients and 291 EGJC patients were identified. Resection was performed in 47 (64.4%), 253 (77.1%), and 212 (72.9%) patients, respectively (*P* = 0.068). 

In GSC patients submitted to resection, 29 had previously been submitted to a Bilroth II anastomosis. Invasion of the previous anastomosis was observed in 31 (66.0%) patients. Splenectomy was performed in 28 (59.6%) and one patient was simultaneously submitted to partial hepatectomy due to local invasion of the left liver.

Table 1 summarizes the clinical and pathological parameters of GSC, PGC, and EGJC. Significant differences were observed in gender (*P* < 0.001): the relative percentage of male patients was higher in GSC than in the other tumours, with a male/female ratio of 14.7 in GSC, 1.8 in PGC, and 2.8 in EGJC. The mean age of GSC patients (67.7 ± 9.8 years) was significantly higher (*P* = 0.014) than the mean age of PGC patients (63.2 ± 12.3 years) or of EGJC patients (62.0 ± 12.1 years). 

The relative frequency of R0 resections observed in our series was higher in GSC than that observed in PGC or in EGJC (*P* = 0.011). 

Regarding tumour variables, there were significant differences when comparing the macroscopic appearance of the considered tumours (*P* = 0.007): the relative frequency of fungating and ulcerofungating carcinomas was higher in GSC than in PGC or in EGJC. No significant differences were observed in the median tumour size: GSC = 5.0 cm, PGC = 5.6 cm, and EGJC = 6.0 cm, *P* = 0.08. Significant differences were detected when tumours were divided according to Laurén classification (*P* = 0.008): the relative frequency of diffuse type was higher in PGC than in GSC or in EGJC. Lymphatic invasion was less frequent in PGC than in GSC or in EGJC (*P* = 0.004), and no significant differences were found regarding venous invasion. 

In this series, significant differences were observed according to the parameters used to stage the carcinomas. Considering depth wall penetration, the relative frequency of pT1 cases was higher in PGC (*P* = 0.003) than in the other tumours. 

The median number of lymph nodes retrieved in GSC patients was 12.0 (1–39), which was significantly lower (*P* = 0.013) than that in PGC (24.0) or in EGJC patients (23.0). Considering all patients submitted to resection, only 18 (38.3%) patients with GSC had fifteen or more lymph nodes removed during lymphadenectomy, contrasting with 210 (83.0%) PGC and 165 (77.8%) EGJC patients that received appropriate lymphadenectomy. The median number of positive nodes was not statistically different in the three types of carcinomas evaluated. Additionally, no significant differences were observed in the distribution of cases according to the nodal status. Nevertheless, the distribution of cases according to the pN categories was significantly different among the three types of tumours (*P* = 0.013). In this study the percentage of pN3 cases was lower in GSC than in the other tumours.

No differences were observed in the distribution of cases according to the presence of distant metastasis. Grouped into stages, GSC and PGC patients were catalogued into lower stages than EGJC patients, but the differences observed were not significant (*P* = 0.076).

**Figure 1:**
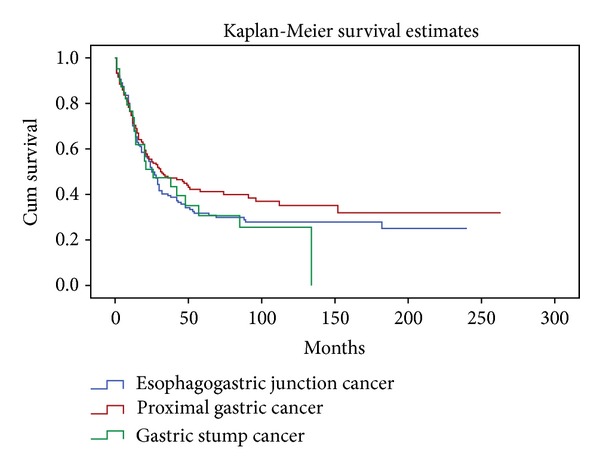
Cumulative survival according to the location of the tumour (*P* = ns).

Five year cumulative survival was 30.7% in GSC, 41.2% in PGC, and 31.7% in EGJC. Median survival time was 26 months (95% CI: 3.4–48.6) in GSC, 31 months (95% CI: 15.2–46.8) in PGC, and 25 months (95% CI: 20.4–29.6) in EGJC. Cumulative survival curves of GSC, PGC and EGJC patients submitted to R0 resection are illustrated in Figure 1.

Table 2 summarizes the univariate analysis performed in this series. Significant differences were observed in the cumulative survival of GSC patients according to the Laurén classification (*P* = 0.006), but no significant differences were observed according to other parameters evaluated, namely, pT, pN, or pTNM stages. No significant survival differences were observed when cases of GSC with or without invasion of previous anastomosis were compared (median of survival 20 and 57 months, resp.) (*P* = 0.117).

The differences detected in cumulative survival observed in patients with the other types of tumours (PGC and EGJC) are also documented in Table 2 and can be compared to GSC. 

Table 3 summarizes the results of the multivariate analysis performed to identify prognostic factors in the whole series. Lymph node status was excluded from this analysis. Cox regression model identified macroscopic appearance (*P* < 0.001), depth of wall penetration (pT) (*P* < 0.001), and venous invasion (*P* = 0.012) as the independent prognostic factors in the global series. The model failed to identify tumour location (i.e., GSC, PGC, or EGJC) as a statistically significant prognostic factor.

## 4. Discussion

It is widely accepted that the biological behavior of gastric cancer is dependent on its proximal or distal location within the stomach [7, 12]. Zhang et al. [13], in a retrospective analysis of 2613 gastric cancer patients, identified upper third location as a factor independently correlated with poor survival. This may be due to rich lymphatic drainage around the esophagogastric junction or to late diagnose at advanced stages with esophageal invasion [14, 15]. 

Although being a proximal cancer, GSC presents some theoretical differences because carcinogenesis is possibly related to local changes induced by previous partial gastric resection for PUD. Probably both the enterogastric reflux of biliopancreatic juice and the hypochlorhydria due to vagotomy are the mechanisms responsible for chronic damage to the remnant mucosa. These hypothetical causes are supported by some studies [2, 16] demonstrating that the great majority of GSCs arise after Billroth II reconstructions and intersects the anastomosis—such findings were observed in our series. These processes cannot be implicated in the other types of proximally located gastric cancers, and therefore they give rise to the question whether GSC has a unique biological behavior. 

The clinical presentation of GSC patients might be very similar to postgastrectomy syndromes. The symptoms can be neglected both by patients and by clinicians [17], which may be one of the reasons why GSC patients are usually diagnosed at advanced stages. Our results are in accordance with this statement, as more than half of our GSC patients are diagnosed in stage III/IV. In an attempt to enhance the chances of early diagnosis of GSC, annual endoscopic screening should be started at least 10 years after partial gastrectomy for PUD [18], and clinicians should keep a low threshold in suspecting cancer whenever new symptoms are found in these patients. 

Male patients have an increased risk for both PUD and for gastric cancer. As expected, in our series, most GSC patients were males, which was also reported by others [19]. We also observed that GSC patients were older: this is related to the long latency period required between previous surgery and the development of these tumours [20]. Even though age is not considered a prognostic factor, survival rate after curative gastrectomy in elderly patients may be lower than that of younger patients. Increased comorbidities and poorer nutritional status are responsible for more non-cancer-related deaths in the elderly patients [21]. 

Previous gastric resection tends to render surgery for GSCs more challenging, due to adhesions and anatomical changes [22]. Nevertheless, we have not found significant differences in the resectability rates among GSC, PGC, and EGJC. Interestingly R0 resections, which are known to be an important prognostic factor [23, 24], were higher in patients with GSC than in PGC or EGJC patients. 

The assessment of the pathologic parameters revealed significant differences according to the classification of Laurén: GSC and EGJC shared a similar pattern with a higher percentage of cases categorized as intestinal-type carcinomas, and PGC displayed a higher percentage of diffuse-type carcinomas. Thorban et al. [24] did not report these differences. 

The distribution of cases according to depth of wall penetration was also different between groups, with more advanced cases in GSC. As 66% of GSC invaded previous anastomosis, we may question if depth wall penetration of GSC is facilitated by the presence of a previous gastroenterostomy. Hypothetic reasons could be (1) increased exposure to bile, (2) altered local inflammatory response, or (3) poorer local irrigation. Furthermore, in our results, the cumulative survival of patients with GSC was not significantly different according to the pT stage of the TNM staging system, contrasting with PGC and EGJC patients, which may reflect the complex pattern of depth wall penetration related to the presence of a previous anastomosis. Nonetheless our results have not revealed any survival differences regarding tumour invasion of previous anastomosis. There is some controversy regarding this topic as some studies [18, 23] have demonstrated survival advantages for tumours located at previous anastomosis, while others [24, 25] have not corroborated these findings. 

Previous studies [26] reported that cancers originated in the gastric stump have a unique pattern of lymph node metastasis, probably related to previous surgery. Han et al. [22] demonstrated that 17% of patients who underwent resection had lymph node metastasis within the jejunal mesentery, beyond the gastrojejunal anastomosis. For these reasons, an en bloc resection of the jejunal mesentery and a cautious lymphadenectomy must be considered if an R0 resection is to be achieved in GSC. 

In the present series, the number of retrieved lymph nodes was significantly lower in cases of GSC compared to that observed in PGC or in EGJC. This difference may be explained by the previous resection, resulting in more adverse surgical conditions, or to the aforementioned altered lymphatic spread [26]. The presence of nodal metastases and the nodal ratio were not different between groups but the analysis of the nodal status of the TNM staging system identified significant differences. Indeed, as expected by the inferior number of retrieved lymph nodes in cases of GSC, the group classified as pN3 was less frequent in GSC patients. In our opinion this finding represents an understaging of GSC cases, due to fewer nodes retrieved.

Overall cumulative survival was not different between GSC, PGC, and EGJC patients. These results are in agreement with other studies [23, 24, 27] and essentially means that the considered groups are proximally located gastric cancers, therefore carrying a dismal prognosis when compared to more distal lesions. Unlike the stratification that TNM staging system has on cumulative survival of PGC and EGJC patients, in our series we found no such effect on GSC cases. This lack of association might be linked to some of the aforementioned particular aspects of GSC, concerning both gastric wall depth invasion and also the different pattern of lymphatic spread. Although no significant differences regarding the presence of nodal metastasis or nodal ratio were observed, it was also documented that the absence of nodal involvement was not related to survival advantages in GSC cases, in contrast to PGC and EGJC patients. These observations raise the question about the suitability of the present TNM staging system to predict outcomes of GSC patients. Therefore, based on the results observed in our series, the N stage of 7th edition of TNM staging system seems unsuitable for GSC patients, which has also been claimed by Li et al. [28].

We must stress that our study has a relatively small number of GSC patients submitted to surgery (as the majority of the published series related to this subject) when compared to PGC and EGJC cases, which may justify some of the statistical disparities observed between groups. Further prospective studies are needed to clarify our findings. 

In conclusion, although some differences in clinicopathologic parameters were observed in GSC when compared to other proximal gastric cancers, the survival rates were similar and consistent with the proximal location of these tumours. Accordingly, the same surgical strategy, which is aimed for a radical R0 resection with adequate lymphadenectomy, should be proposed for all proximally located gastric cancers. GSC cannot be exactly evaluated by using the TNM classification for gastric cancer, owing to the interfering of previous operation. Some particular aspects, namely, the altered pattern of lymph node metastasis, seem to be of utmost relevance in gastric stump cancers and should, in our opinion, call for the design of more suitable staging systems for these specific carcinomas.

## Figures and Tables

**Table 1:** Distribution of clinicopathologic parameters according to the type of carcinoma.

Parameters	GSC *n* = 47 (9.2)	PGC *n* = 253 (49.4)	EGJC *n* = 212 (41.4)	*P* value
Gender				**<0.001**
Male	44 (93.6)	164 (64.8)	157 (74.1)	
Female	3 (6.4)	89 (35.2)	55 (25.9)	
Resection status				**0.011**
R0	42 (89.4)	152 (83.1)	70 (70.7)	
R1/R2	5 (10.6)	31 (16.9)	29 (29.3)	
Macroscopy				**0.007**
Fungat./ulcerofungat.	24 (52.2)	76 (32.2)	83 (43.0)	
Ulcerating/ulceroinfilt.	15 (32.6)	140 (59.3)	91 (47.2)	
Infiltrative	7 (15.2)	20 (8.5)	19 (9.8)	
Lauren classification				**0.008**
Intestinal	25 (55.6)	106 (44.2)	112 (58.6)	
Diffuse	5 (11.1)	57 (23.8)	24 (12.6)	
Unclassified	15 (33.3)	77 (32.1)	55 (28.8)	
Depth of wall penetration				**0.003**
pT1	3 (6.5)	48 (19.6)	16 (7.9)	
pT2	5 (10.9)	20 (8.2)	26 (12.9)	
pT3	22 (47.8)	90 (36.7)	70 (34.7)	
pT4	16 (34.8)	87 (35.5)	90 (44.6)	
Nodal metastases				**0.49**
Absent	17 (36.2)	82 (35.7)	53 (30.3)	
Present	30 (63.8)	148 (64.3)	122 (69.7)	
Nodal metastases				**0.013**
pN0	17 (36.2)	82 (35.7)	53 (30.3)	
pN1	11 (23.4)	40 (17.4)	31 (17.7)	
pN2	13 (27.7)	28 (12.2)	38 (27.7)	
pN3	6 (12.8)	80 (34.8)	53 (30.3)	
Nodal ratio				**0.48**
≤20%	27 (57.4)	140 (61.7)	92 (55.8)	
>20%	20 (42.6)	87 (38.3)	73 (44.2)	
Lymphatic vessels invasion				**0.004**
Absent	6 (12.8)	61 (25.3)	28 (13.6)	
Present	41 (87.2)	180 (74.7)	178 (86.4)	
Venous invasion				**0.26**
Absent	19 (40.4)	109 (45.0)	76 (37.4)	
Present	28 (59.6)	133 (55.0)	127 (62.6)	
Distant metastasis				**0.39**
Absent	45 (95.7)	225 (90.0)	188 (92.2)	
Present	2 (4.3)	25 (10.0)	16 (7.8)	
AJCC stage				**0.076**
I-II	21 (45.7)	111 (48.9)	65 (37.6)	
III-IV	25 (54.3)	116 (51.1)	108 (62.4)	

Values in parentheses are percentages unless indicated otherwise.

**Table 2:** Univariate analysis of patients' survival with GSC, PGC, and EGJC, after R0 resection.

Parameters	GSC	PGC	EGJC
Gender	*P* = 0.960	*P* = 0.340	*P* = 0.189
Macroscopy	*P* = 0.050	***P*** = **0**.**001**	***P*** = **0**.**006**
Lymphatic vessels invasion	*P* = 0.176	***P*** = **0**.**001**	*P* = 0.069
Venous invasion	*P* = 0.398	***P*** = **0**.**007**	***P*** = **0**.**008**
Lauren classification	***P*** = **0**.**006**	***P*** = **0**.**002**	*P* = 0.607
Depth wall penetration (T stage)	*P* = 0.220	***P*** < **0**.**001**	***P*** = **0**.**025**
Lymph node metastases (present versus absent)	*P* = 0.135	***P*** < **0**.**001**	***P*** = **0**.**004**
Nodal metastases (N stage)	*P* = 0.496	***P*** < **0**.**001**	***P*** = **0**.**002**
Nodal ratio (≤20% versus >20%)	*P* = 0.057	***P*** < **0**.**001**	***P*** < **0**.**001**
AJCC stage grouped (I-II versus III-IV)	*P* = 0.489	***P*** < **0**.**001**	***P*** = **0**.**004**

**Table 3:** Summary of Cox regression analysis identifying prognostic factors in the whole series.

Parameters	HR	HR 95% IC	*P* value
Macroscopic appearance	1.48	1.20–1.81	<0.001
pT	1.28	1.13–1.45	<0.001
Venous invasion	1.43	1.08–1.88	0.012
